# Hypokalaemic paralysis and metabolic alkalosis in a patient with Sjögren syndrome: a case report and literature review

**DOI:** 10.1186/s12882-021-02371-5

**Published:** 2021-04-30

**Authors:** Rasika Ranaweerage, Shehan Perera, Aruna Gunapala

**Affiliations:** General medicine, National Hospital of Sri Lanka, Colombo, Sri Lanka

**Keywords:** Acquired Gitelman syndrome, Sjögren syndrome, Hypokalaemia, Metabolic alkalosis

## Abstract

**Background:**

Acquired Gitelman syndrome is a very rare disorder reported in association with autoimmune disorders, mostly Sjögren syndrome. It is characterized by the presence of hypokalaemic metabolic alkalosis, hypocalciuria, hypomagnesaemia and hyper-reninaemia, in the absence of typical genetic mutations associated with inherited Gitelman syndrome.

**Case presentation:**

A 20 year old woman who was previously diagnosed with primary Sjögren syndrome and autoimmune thyroiditis presented with two week history of lower limb weakness and salt craving. Examination revealed upper limb and lower limb muscle weakness with muscle power of 3/5 on MRC scale and diminished deep tendon reflexes. On evaluation, she had hypokalaemia with high trans-tubular potassium gradient, metabolic alkalosis and hypocalciuria, features suggestive of Gitelman syndrome. New onset hypokalaemic alkalosis in a previously normokalaemic patient with Sjögren syndrome strongly favored a diagnosis of acquired Gitelman syndrome. Daily potassium supplementation and spironolactone resulted in complete clinical recovery.

**Conclusions:**

Acquired Gitelman syndrome associated with Sjögren syndrome is rare. It should be considered as a differential diagnosis during evaluation of acute paralysis and hypokalaemic metabolic alkalosis in patients with autoimmune disorders, especially Sjögren syndrome.

**Supplementary Information:**

The online version contains supplementary material available at 10.1186/s12882-021-02371-5.

## Background

Sjögren syndrome is a systemic autoimmune disease primarily affecting the exocrine glands. Renal involvement in Sjögren syndrome usually manifest as chronic interstitial nephritis, distal renal tubular acidosis and nephrogenic diabetes insipidus. Sjögren syndrome presenting with features of Gitelman syndrome is rare and named as acquired Gitelman syndrome. In medical literature only eight such cases have been reported. Awareness of the treating physician regarding this rare clinical presentation is imperative to avoid fatal outcomes, especially in patients presenting with acute paralysis. Here, we describe a young female with Sjögren syndrome presenting with features of Gitelman syndrome and hypokalaemic paralysis.

## Case presentation

A 20 year old woman previously diagnosed with primary Sjögren syndrome and autoimmune thyroiditis with hypothyroidism presented with two week history of muscle cramps, gradual onset, progressive lower limb weakness and salt craving. The weakness was not associated with ingestion of high carbohydrate meals, diurnal variation of symptoms, easy fatigability or other neurological symptoms including diplopia, bulbar involvement and paresthesia. There was no preceding respiratory tract infection or gastroenteritis. She was on Thyroxin 50 µg daily and denied the use of diuretics or laxatives. There was no personal or family history of similar episodes. Her blood pressure was 100/70 mmHg. Pulse rate was 78 bpm. She was not volume depleted. There was no apparent wasting or tenderness of muscles. Muscle power was 3/5 on MRC scale. Deep tendon reflexes were diminished. Rest of the clinical examination was normal.

Her investigation results revealed that she was having hypokalaemia. ECG revealed T wave flattening and U waves. Imaging did not reveal nephrocalcinosis. Her investigation results are summarized in Table 1.

**Table 1:** Summary of laboratory investigations

Investigation	Result	Normal range
Haemoglobin	12 g/dL	12.0–15.5 g/dL
Platelets	200,000 per microL	150–450 per microL
**Serum investigations**		
Sodium	135 mmol/L	135–145 mmol/L
Potassium	2.5 mmol/L	3.5–5 mmol/L
Magnesium	1.8 mg/dL	1.7–2.30 mg/dL
Chloride	91 mmol/L	95–111 mmol/L
Creatinine	0.8 mg/dL	0.6–1.2 mg/dL
Arterial pH	7.5	7.35–7.45
PaCO_2_	43 mmHg	38–42 mmHg
Bicarbonate	28 mmol/L	22–28 mmol/L
Renin activity	60.6 ng/mL/h	5.4–34.5 ng/mL/h
TSH	3.872 mIU/L	0.5 to 5.0 mIU/L
Free T_4_	1.29 ng/dL	0.9–2.3 ng/dL
9 a.m. Serum cortisol	550 nmol/L	140 to 690 nmol/L
**Urinary investigations**		
Urinary potassium	40 mmol/L	< 20 mmol/L
Urinary chloride	64 mmol/L	55–125 mmol/L
Trans-Tubular Potassium Gradient (TTKG)	13	< 3
Spot urine potassium/creatinine ratio	3.1 mmol/mmol	> 2 mmol/mmol
Spot urine calcium/creatinine ratio	0.01 g calcium /g creatinine	< 0.2 g calcium /g creatinine

Previous records revealed that she was diagnosed with primary Sjögren syndrome and autoimmune thyroiditis 2 years ago, when she presented with salivary gland swelling and sicca symptoms. She did not have any other symptoms and signs suggestive of connective tissue disorders. Salivary gland biopsy showed periductal and periacinic lymphoplasmacytic infiltrate that was compatible with Sjögren syndrome. Rheumatoid factor ,anti-Ro (SSA) antibody, anti TPO antibody and Thyroglobulin antibody were positive. ANA titer was positive at 1:320. At that time, serum potassium was noted to be within normal parameters.

Presence of symptomatic hypokalemia with high Trans-tubular Potassium Gradient (TTKG) was suggestive of renal loss of potassium. Coexisting metabolic alkalosis, absence of hypercalciuria, increased renin activity with normal blood pressure in the absence of diuretic or laxative use favored a diagnosis of Gitelman syndrome, either inherited or acquired. Previous records revealed persistent normokalaemia prior to this presentation. New onset hypokalaemic alkalosis in a previously normokalaemic patient with Sjögren syndrome (which is frequently associated with the renal tubular dysfunctions) and autoimmune thyroiditis strongly favored acquired Gitelman syndrome of immune origin. Genetic studies are crucial in differentiating between the inherited and acquired forms of Gitelman syndrome, but we did not have access to genetic studies to confirm our diagnosis.

She was treated with intravenous and oral potassium salts which resulted in improvement of symptoms. At discharge, maintenance potassium salts and spironolactone were prescribed which she tolerated well. Two months later she had a relapse due to poor compliance. After ensuring the compliance she had no further relapses and remained symptoms free thereafter.

## Discussion and conclusion

Primary Sjögren syndrome is a systemic autoimmune disease characterized by lymphoplasmacytic infiltration of lacrimal and salivary glands and multiple extraglandular tissues including skin and joints, lungs, heart, gastrointestinal tract, kidneys, bladder, gynecological system and nervous system [1, 2]. It is commonly seen in females with typical onset in fourth to fifth decade of life. The diagnosis of Sjögren syndrome is made in the presence of typical clinical signs, serological and/or histological evidence after the exclusion of conditions that may manifest similar to Sjögren syndrome [3].

Renal involvement is a frequent manifestation of Sjögren syndrome. It is rarely overt and may precede onset sicca symptoms [4]. The manifestations vary from chronic interstial nephritis, distal tubular acidosis, and nephrogenic diabetes insipidus to symptomatic hypokalaemia due to tubular injury [4, 5]. The commonest presentation is chronic interstial nephritis [6]. Distal renal tubular acidosis (RTA) may occur in up to 25 % of the patients with Sjögren syndrome. It is characterized by normal anion gap metabolic acidosis and urinary potassium wasting leading to hypokalaemia muscle paralysis with respiratory arrest which has been the presenting symptom in some cases of Sjögren syndrome. Glomerular involvement is noted to be rare and carries high mortality [4].

Gitelman syndrome is a renal tubular disorder characterized by hypokalemia, metabolic alkalosis, hypomagnesaemia, hypocalciuria, and hyper-reninaemia with normal blood pressure. Gitelman syndrome is an autosomal recessively inherited genetic disorder caused due to the biallelic inactivating mutations of SLC12A3 gene encoding for thiazide-sensitive Sodium chloride cotransporter (NCCT) in distal tubule of the kidney. Gitelman syndrome is usually not diagnosed until late childhood or adulthood [7]. More common disorders that have a similar presentation and must be excluded are surreptitious vomiting, surreptitious diuretic use and hypokalaemic periodic paralysis.

A rare, phenotypically similar clinical presentation has been described in patients with autoimmune disorders such as Sjögren syndrome, Systemic lupus erythematosus and Systemic sclerosis, which has been named as “acquired Gitelman syndrome” [8–11]. There are only eight such cases have been reported in patients with primary Sjögren syndrome and three of these patients presented with hypokalemic paralysis [12–15]. It should be considered as a differential diagnosis during evaluation of hypokalaemic metabolic alkalosis in patients with autoimmune disorders, especially Sjögren syndrome.

Pathogenesis of acquired Gitelman syndrome associated with Sjögren syndrome is being evaluated. Kim et al. reported the presence of a circulating autoantibody against NCCT in a patient presenting with acquired Gitelman syndrome secondary to Sjögren syndrome. This was demonstrated by incubation of serum of a patient with acquired Gitelman syndrome with normal mouse kidney tissue, which showed similar staining patterns of NCCT of distal tubules compared to the incubation of normal mouse kidney with rabbit polyclonal anti-NCCT antibody [8, 14]. Genetic studies are crucial in differentiating between the genetic and the acquired form of Gitelman syndrome. In comparison, type 1 renal tubular acidosis (RTA) secondary to Sjögren syndrome was found to be associated with tubular interstial nephritis with lymphocytic infiltration and reduced expression of both anion exchanger 1 (AE1) and hydrogen-ATPase located in the α-intercalated cells in absence of autoantibodies against these transporters [16].In recent studies, a subset of patients with RTA with anti- carbonic anhydrase antibodies has been identified [17]. These antibodies are thought to be contributory to the pathogenesis of RTA in Sjögren syndrome.

Hypomagnesaemia is a characteristic manifestation of classic Gitelman syndrome, but magnesium level can even be normal in some patients [7]. The mechanism of hypomagnesaemia has been attributed to down regulation TRPM6 magnesium-permeable channels that are located at the apical domain of the distal convoluted tubules and brush border of the duodenal magnesium-transporter cells [18]. In our patient magnesium level was normal.

Our patient demonstrated a very good clinical response to potassium supplementation, but she needed very high doses of potassium chloride to maintain normokalaemia. Similar clinical responses to potassium and /or magnesium and/or spironolactone was noted among other cases as well. In five case reports, steroids were administered with above treatment [13–15]. Four patients demonstrated reduced renal potassium wasting while one patient continued to have renal potassium wasting while on steroids. One patient was given cyclophosphamide [19]. None of these patients had relapses while on treatment. Three patients who had apparently responded to steroids were also documented to be on spironolactone at the same time. Hence the observed reduction of renal potassium wasting cannot be attributed to action of steroids alone.

In comparison, the effects of immunosuppression with steroids in type 1 renal tubular acidosis secondary to Sjögren syndrome has been extensively evaluated [20]. Immunosuppression with steroids has been found to effective in slowing the progression of renal disease in majority of these patients. It is our opinion that use of steroids in management of acquired Gitelman syndrome in Sjögren syndrome needs further evaluation as a potential therapeutic modality. Presence of lymphoplasmacytic cell infiltration in renal biopsy may also assist in deciding on immunosuppressive therapy.

Further studies focused on the pathogenesis of renal tubular dysfunctions in Sjögren syndrome will be immensely beneficial in establishing the prognosis and effective therapeutic interventions in this rare and possibly underdiagnosed manifestation of Sjögren syndrome. Paucity of diagnosed patients with acquired Gitelman syndrome appears to be a major limiting factor in studying pathogenesis and treatment responsiveness. Establishing a global disease registry and long term follow up of the diagnosed patients may help to alleviate this issue.

## Supplementary Information

**Additional file 1.**

## Data Availability

All necessary data and material are provided.

## Abbreviations

bpm: beats per minute
MRC scale: Medical research council scale
NCCT: Sodium chloride co-transporter
SLC12A3: Solute carrier family 12 member 3
TRPM6: Transient receptor potential melastatin 6
RTA: Renal tubular acidosis

## References

[1] Ramos-Casals M, Tzioufas AG, Font J. Primary Sjögren’s syndrome: new clinical and therapeutic concepts. Annals of the rheumatic diseases. 2005 Mar 1;64(3):347 – 54. 10.1136/ard.2004.025676 PMC1755414 15498797

[2] ASMUSSEN K, ANDERSEN V, BENDIXEN G, SCHIØDT M. OXHOLM P. A new model for classification of disease manifestations in primary Sjögren’s syndrome: evaluation in a retrospective long-term study. Journal of internal medicine. 1996 Jun;239(6):475–82. 10.1046/j.1365-2796.1996.418817000.x 8656140

[3] Shiboski CH, Shiboski SC, Seror R, Criswell LA, Labetoulle M, Lietman TM, Rasmussen A, Scofield H, Vitali C, Bowman SJ, Mariette X. 2016 ACR-EULAR classification criteria for primary Sjögren’s syndrome: a consensus and data-driven methodology involving three international patient cohorts. Arthritis & rheumatology (Hoboken. 2017 Jan;69(1):p. 35. 10.1002/art.39859 PMC5650478 27785888

[4] Goules A, Masouridi S, Tzioufas AG, Ioannidis JP, Skopouli FN, Moutsopoulos HM. Clinically significant and biopsy-documented renal involvement in primary Sjögren syndrome. Medicine. 2000 Jul 1;79(4):241-9. 10.1097/00005792-200007000-00005 10941353

[5] Nagayama Y, Shigeno M, Nakagawa Y, Suganuma A, Takeshita A, Fujiyama K, Ashizawa K, Kiriyama T, Yokoyama N, Nagataki S. Acquired nephrogenic diabetes insipidus secondary to distal renal tubular acidosis and nephrocalcinosis associated with Sjögren’s syndrome. J Endocrinol Investig. 1994 Sep;17(8):659–63. 10.1007/BF03349682 7868806

[6] Maripuri S, Grande JP, Osborn TG, Fervenza FC, Matteson EL, Donadio JV, Hogan MC. Renal involvement in primary Sjögren’s syndrome: a clinicopathologic study. Clinical Journal of the American Society of Nephrology. 2009 Sep 1;4(9):1423-31. 10.2215/CJN.00980209 PMC2736689 19679669

[7] Blanchard A, Bockenhauer D, Bolignano D, Calo LA, Cosyns E, Devuyst O, Ellison DH, Frankl FE, Knoers NV, Konrad M, Lin SH. Gitelman syndrome: consensus and guidance from a kidney disease: improving global outcomes (KDIGO) controversies conference. Kidney international. 2017 Jan 1;91(1):24–33. 10.1016/j.kint.2016.09.046 28003083

[8] Kim YK, Song HC, Kim YS, Choi EJ. Acquired gitelman syndrome. Electrolyte Blood Press. 2009 Jun;7(1):5–8. 10.5049/EBP.2009.7.1.5 PMC3041481 21468178

[9] Barathidasan GS, Krishnamurthy S, Karunakar P, Rajendran R, Ramya K, Dhandapany G, Ramamoorthy JG, Ganesh RN. Systemic lupus erythematosus complicated by a Gitelman-like syndrome in an 8-year-old girl. CEN case reports. 2019 Dec 18:1–4. 10.1007/s13730-019-00440-1 PMC7148407 31853802

[10] Masab M, Goyal A, Abrol S, Rangaswami J. Acquired Gitelman Syndrome associated with systemic sclerosis. Cureus. 2019 Jan;11(1). 10.7759/cureus.3923 PMC6430306 30931194

[11] Casatta L, Ferraccioli GF, Bartoli E. Hypokalaemic alkalosis, acquired Gitelman’s and Bartter’s syndrome in chronic sialoadenitis. British journal of rheumatology. 1997 Oct 1;36(10):1125-8. 10.1093/rheumatology/36.10.1125 9374934

[12] Chen YC, Yang WC, Yang AH, Lin SH, Li HY, Lin CC. Primary Sjögren’s syndrome associated with Gitelman’s syndrome presenting with muscular paralysis. American journal of kidney diseases. 2003 Sep 1;42(3):586 – 90. 10.1016/s0272-6386(03)00792-3 12955689

[13] Schwarz C, Barisani T, Bauer E, Druml W. A woman with red eyes and hypokalemia: a case of acquired Gitelman syndrome. Wiener klinische Wochenschrift. 2006 May;118(7):239–42. 10.1007/s00508-006-0559-4 16794762

[14] Kim YK, Song HC, Kim WY, Yoon HE, Choi YJ, Ki CS, Park CW, Yang CW, Kim J, Kim YS, Choi EJ. Acquired Gitelman syndrome in a patient with primary Sjögren syndrome. American Journal of Kidney Diseases. 2008 Dec 1;52(6):1163-7. 10.1053/j.ajkd.2008.07.025 18805608

[15] Kulkarni M, Kadri P, Pinto R. A case of acquired Gitelman syndrome presenting as hypokalemic paralysis. Indian J Nephrol. 2015 Jul 1;25(4):246-7. 10.4103/0971-4065.146031 PMC4495481 26199478

[16] Walsh S, Turner CM, Toye A, Wagner C, Jaeger P, Laing C, Unwin R. Immunohistochemical comparison of a case of inherited distal renal tubular acidosis (with a unique AE1 mutation) with an acquired case secondary to autoimmune disease. Nephrology Dialysis Transplantation. 2007 Mar 1;22(3):807 – 12. 10.1093/ndt/gfl662 17205967

[17] Pertovaara M, Bootorabi F, Kuuslahti M, Pasternack A, Parkkila S. Novel carbonic anhydrase autoantibodies and renal manifestations in patients with primary Sjögren’s syndrome. Rheumatology. 2011 Aug 1;50(8):1453-7. 10.1093/rheumatology/ker118 21427176

[18] McCormick JA, Ellison DH. Distal convoluted tubule. Comprehensive Physiology. 2011 Jan 17;5(1):45–98. 10.1002/cphy.c140002 PMC5810970 25589264

[19] Gu X, Su Z, Chen M, Xu Y, Wang Y. Acquired Gitelman syndrome in a primary Sjögren syndrome patient with a SLC12A3 heterozygous mutation: a case report and literature review. Nephrology. 2017 Aug;22(8):652–5. 10.1111/nep.13045 PMC6099516 28685938

[20] Kaufman I, Schwartz D, Caspi D, Paran D. Sjögren’s syndrome–not just Sicca: renal involvement in Sjögren’s syndrome. Scandinavian journal of rheumatology. 2008 Jan 1;37(3):213-8. 10.1080/03009740701867323 18465457

