# Supplementary material for: Hypokalaemic paralysis and metabolic alkalosis in a patient with Sjögren syndrome: a case report and literature review
Source: BMC Nephrol. 2021 Apr 30;22:159. doi: 10.1186/s12882-021-02371-5 (PMC8086307; doi:10.1186/s12882-021-02371-5)
Supplement: Supplementary file 1 — Additional file 1. [file 12882_2021_2371_MOESM1_ESM.docx]

**Timeline**

Diagnosed with primary Sjögren syndrome and autoimmune thyroiditis.

Admission - Presented with weakness of upper and lower limbs and salt craving for two weeks duration

07^th^ January 2020

March 2018

Hypokalaemia with high trans-tubular potassium gradient, metabolic alkalosis and hypocalciuria

Regained normokalaemia and improved muscle weakness.

Discharged.

12^th^ January 2020

**Potassium supplementation**

Two months later relapsed due to poor compliance to potassium supplementation.

With counselling and good compliance to treatment now she is relapse free for a period of one year.
